# Efficient Iodine Removal by Porous Biochar-Confined Nano-Cu_2_O/Cu^0^: Rapid and Selective Adsorption of Iodide and Iodate Ions

**DOI:** 10.3390/nano13030576

**Published:** 2023-01-31

**Authors:** Jiaqi Li, Mengzhou Wang, Xu Zhao, Zitong Li, Yihui Niu, Sufeng Wang, Qina Sun

**Affiliations:** Hebei Key Laboratory of Heavy Metal Deep-Remediation in Water and Resource Reuse, School of Environmental and Chemical Engineering, Yanshan University, Qinhuangdao 066004, China

**Keywords:** nanoparticles, cuprous, biochar, *Artemia*, iodide ion, iodate ion

## Abstract

Iodine is a nuclide of crucial concern in radioactive waste management. Nanomaterials selectively adsorb iodine from water; however, the efficient application of nanomaterials in engineering still needs to be developed for radioactive wastewater deiodination. *Artemia* egg shells possess large surface groups and connecting pores, providing a new biomaterial to remove contaminants. Based on the *Artemia* egg shell-derived biochar (AES biochar) and in situ precipitation and reduction of cuprous, we synthesized a novel nanocomposite, namely porous biochar-confined nano-Cu_2_O/Cu^0^ (C-Cu). The characterization of C-Cu confirmed that the nano-Cu_2_O/Cu^0^ was dispersed in the pores of AES biochar, serving in the efficient and selective adsorption of iodide and iodate ions from water. The iodide ion removal by C-Cu when equilibrated for 40 min exhibited high removal efficiency over the wide pH range of 4 to 10. Remarkable selectivity towards both iodide and iodate ions of C-Cu was permitted against competing anions (Cl^−^/NO_3_^−^/SO_4_^2−^) at high concentrations. The applicability of C-Cu was demonstrated by a packed column test with treated effluents of 1279 BV. The rapid and selective removal of iodide and iodate ions from water is attributed to nanoparticles confined on the AES biochar and pore-facilitated mass transfer. Combining the advantages of the porous biochar and nano-Cu_2_O/Cu^0^, the use of C-Cu offers a promising method of iodine removal from water in engineering applications.

## 1. Introduction

Nuclear power produces about 10% of the total global electricity and about 5% of the total in China, and it will continue to be a viable alternative to fossil fuels in the future [1]. While nuclear power contributes to electricity supply and reduces CO_2_ emissions, it also poses significant challenges in terms of radioactive waste treatment and disposal. The radionuclides produced by nuclear reaction processes such as in uranium fission mainly contain ^129/131^I, ^127^Xe, ^134/137^Cs, ^90^Sr, ^99^Tc, and ^79^Se [1]. Among these, iodine is particularly problematic due to its volatility. Before treatments involving high temperatures such as vitrification, the liquidus iodine needs to be captured efficiently to avoid the release of gaseous wastes [2]. Additionally, the emergency accident in Fukushima, Japan, released radioactive iodine into the water and soil [3,4]. Radioactive iodine poses a significant threat to living creatures, including humans, and is considered one of the most dangerous radionuclides. As one of the halogen elements, iodine is highly soluble and mobile in terms of its chemical properties. It can quickly enter the human body through the food chain and can cause damage due to its chemical toxicity and radioactivity. Therefore, it is necessary to develop efficient methods to remove radioactive iodine.

Among the different halide removal treatments, adsorption still attracts significant interest because of its operational simplicity and flexibility [5,6]. Activated carbon removes gaseous iodine via physical adsorption, and the ion exchange resin enriches iodine in the form of liquidus ions [7,8]. In recent years, metals such as copper, silver, and bismuth have had a special selective adsorption capacity for iodine [9,10]. Among them, Cu(I)- and Cu(I)-based composite materials are the most promising owing to their high removal efficiency for aqueous iodide anions and their economic cost-effectiveness. Cu(I)- and Cu(I)-based composites remove iodide ions from the water via different mechanisms. In acidic solutions, the cuprous cation Cu^+^ and iodide anion I^−^ produce insoluble CuI precipitates, while in neutral and basic solutions, the iodide anions exchange with the hydroxyl group of Cu-OH and form an inner sphere–surface complex [10]. Moreover, the transient Cu(I) species can be generated by combining Cu(II)-containing compounds and monomeric copper Cu^0^ [11]. However, several issues inhibit the direct use of Cu(I) oxide or compounds for iodine removal from water. First, in neutral and acidic water, Cu^+^ is extremely unstable under oxidizing conditions and undergoes complex chemical reactions that change the valence state and affect the removal of iodine. Secondly, the oxidation states of iodine, mainly for iodate anions (IO_3_^−^) in natural water, mean it is difficult for it to adsorb on solids based on Cu(I) [12]. Thirdly, the solvability of Cu^+^ may be problematic in water because copper can be very toxic when ingested in excess and is also highly toxic to aquatic life [13]. These issues are the constraints that affect the selective removal of iodine from water using copper-based materials.

In addition to the concern regarding the selectivity in the iodine removal process, the fast kinetic properties are also the focus of iodine removal materials. This is due to the differences in the hazardous effects of the different iodine isotopes on the environment and people. There are 24 radioactive isotopes of iodine with different adverse effects on the loss of control in the environment. The most typical radioactive iodine is ^129^I, which has an extremely long half-life of 1.57 × 10^7^ years [14]. Its environmental risk is persistent and receives much attention in radioactive iodine removal. In contrast, the half-life of the radioactive isotope ^131^I is only 8.02 days, so in some cases it is not considered a long-term hazard [15]. However, the high radioactivity of ^131^I at 4.6 × 10^15^ Bq/g makes it a particularly acute risk to human health [2,16]. Thus, it is crucial to remove radioactive iodine quickly via emergency treatments; unfortunately, this has not been fully emphasized.

Porous materials are traditionally used in adsorption. Conventional activated carbon molecules are widely used for iodine adsorption because of their high surface area, large porosity, good thermal and chemical stability, and potential for facile modification. Additionally, compared to the developing synthetic adsorbents such as porous organic polymers (POPs) and metal–organic frameworks (MOFs), activated carbons are cheaper and simpler to prepare with higher productivity rates [3]. Therefore, the use of porous carbon materials is industrially feasible for iodine enrichment at the present stage. However, the use of low-cost porous structures to provide efficient and selective binding sites for the rapid adsorption of both iodide and iodate ions in water remains a challenge. Sorbent materials should have a unique morphology, the absence of any blocked bulk volume, low energy and overall costs, and high chemical durability. Natural biosubstrates have drawn wide attention for their high porosity, large surface areas, functional groups, and low costs. As an emerging adsorbent and nanoparticle host, *Artemia* egg shell-based composites have proven effective in the enrichment of phosphate, fluoride, and lead, as well as in the degradation of organic contaminants as catalysts [17,18,19,20,21,22,23,24,25,26]. The hierarchical porous macropore–mesopore structure facilitates nanoparticle dispersion and contaminant mass transfer. Thus, the biochar derived from *Artemia* egg shells may stably disperse Cu(I)-containing nanoparticles, offering the possibility of rapid iodine removal from water bodies. To the best of our knowledge, such research has not been reported.

Herein, we synthesized a novel porous nanocomposite noted as C-Cu via the in situ precipitation and reduction of Cu_2_O and Cu^0^ onto a biomatrix, namely *Artemia* egg shell-derived biochar (AES biochar). The iodine removal performance by the C-Cu was investigated through a series of adsorption experiments with iodide and iodate ions. The engineering applicability of C-Cu was evaluated using a fixed-bed column test. The advantageous properties of the porous biochar and nano-Cu_2_O/Cu^0^ are promising for real applications involving iodine removal by nanoparticles.

## 2. Materials and Methods

### 2.1. Materials and Chemicals

The Cu(NO_3_)_2_⋅3H_2_O was purchased from Aladdin Reagent Co., Shanghai, China. The KI, NaI, NaIO_3_, Na_2_SO_4_, NaCl, and NaNO_3_ were purchased from Kemio Chemical Reagent Co., Tianjin, China. The polyethylene imine (99% pure, MW 1800) was purchased from Ron Reagent Co., Shanghai, China. The commercial Cu_2_O was purchased from Sinopharm Chemical Reagent Co., Beijing, China. All chemicals were used without any further purification. The *Artemia* egg shells were provided by the Fisheries Research Institute of Qinhuangdao, Qinhuangdao, China.

### 2.2. Synthesis of Artemia Egg Shell Biochar-Hosted Nano-Cu_2_O/Cu^0^

As the raw material of the biochar, *Artemia* egg shells were washed with deionized water to remove salt and impurities. The washed *Artemia* egg shells were calcinated under vacuum at 550 °C for 3 h with a nitrogen flow rate of 60 mL/min, and the resulting product was *Artemia* egg shell biochar, noted as AES biochar. The Cu^+^ precursor solution was prepared with 1.06 g of polyethyleneimine (PEI) and 1.52 g of Cu(NO_3_)_2_⋅3H_2_O dissolved in 100 ml of deionized water. The Cu^+^ precursor solution and 1.0 g of AES biochar were vigorously mixed by stirring of 12 h. The mixture was then transferred to a 200 mL tetrafluoroethylene hydrothermal reactor and heated hermetically at 220 °C for 2 h. The solid was then filtered and dried at 60 °C to obtain *Artemia* egg shell biochar-hosted nano-Cu_2_O/Cu^0^, denoted as C-Cu. The copper concentration in the filtrate was determined via atomic absorption spectrometry (AAS, AA6800, Shimadzu, Kyoto, Japan) to calculate the copper content of the C-Cu.

### 2.3. Characterization

The morphology and structure of the adsorbents were recorded using a scanning electron microscope (SEM, S-3400N II, Hitachi, Tokyo, Japan) and transmission electron microscope (TEM, JEM-2010FX, JEOL, Tokyo, Japan). The specific surface area (SSA) and pore size distribution of the C-Cu and AES biochar were determined via N_2_ adsorption/desorption with a surface area and pore size analyzer (ASAP 2420, Micromeritics, Norcross, GA, USA). The specific surface areas were calculated from the Brunauer– Emmett–Teller (BET) equation. The total pore volumes were estimated using the adsorbed N_2_ amount at the relative pressure P/P_0_ of 0.99, and the micropore volumes were calculated from the t-plot method. The pore size distribution was obtained based on the Barrett–Joyner–Halenda (BJH) method. X-ray diffraction (XRD) data for the Cu_2_O, AEC biochar, and Cu-C were collected using an X-ray diffractometer (D/max2550PC, Rigaku, Tokyo, Japan) at a scanning speed of 5° 2*θ*/min over a range of 10° to 100°. The zeta potentials of the C-Cu, AES biochar, and Cu_2_O were determined at different pH values using a Zetasizer (Nano ZS-90) from Malvern Instruments, Malvern, UK.

### 2.4. Iodine Adsorption

In the iodine adsorption experiments, stable isotopes of ^127^I was used instead of radioactive iodine ^129/131^I. Solutions containing I^−^ or IO_3_^−^ were prepared with sodium iodide or sodium iodate, respectively. Batch experiments were used for static adsorption, and the C-Cu was placed into solutions with 10 mg/L of I^−^ or IO_3_^−^ at pH = 7.0 ± 0.2. The adsorption system was maintained at 25 °C using a thermostatic shaker with an agitation speed of 180 rpm. The dosage of C-Cu during the kinetic initiation process was 1.0 g and the solution volume was 1000 mL. As the adsorption process continued to various time points, supernatant samples were taken and analyzed using a spectrophotometer for the concentrations of I^−^ or IO_3_^−^ [27]. In the isotherm experiments, 50 mL I^−^ or IO_3_^−^ solutions with different initial concentrations of 1, 5, 10, 20, 50, 100, and 200 mg/L were contacted with 0.025 g of C-Cu for 24 h to allow adsorption equilibrium to be achieved. The pH of the solutions was adjusted to different values ranging between 2 and 12 to investigate the effect of the solution pH on the iodide and iodate ion adsorption onto the C-Cu. Na_2_SO_4_, NaCl, and NaNO_3_ were used in the selective adsorption experiments, and the competing anions of SO_4_^2−^, Cl^−^, and NO_3_^−^ had molar ratios of 1, 5, 10, 20, 50, and 100 mol/mol. Cu_2_O and AEC biochar were used as the reference materials for the static adsorption experiments.

The column adsorption experiment was conducted to evaluate the working conditions of the adsorbents in the fixed-bed reactor to investigate the removal efficiency in engineering applications. A column with a 10 mm inner diameter was packed with 1.5 g of C-Cu to form a 25-mm-high fixed bed. The influent-simulated radioactive wastewater was percolated into the column at a constant flow rate using a peristaltic pump containing I^−^ = 2 mg/L, Cl^−^ = 150 mg/L, SO_4_^2−^ = 50 mg/L, NO_3_^−^ = 50 mg/L, F^−^ = 50 mg/L, ReO_4_^−^ = 10 mg/L, and HCO_3_^−^ = 50 mg/L. An automatic collector collected the effluent samples for the iodide ion concentration measurements.

## 3. Results and Discussion

### 3.1. Characterization

**Figure 1:**
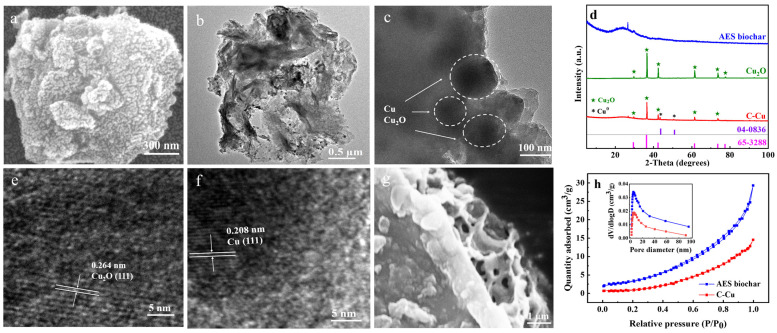
(**a**) SEM image and TEM images of C-Cu at (**b**) 0.5 μm and (**c**) 100 nm. (**d**) XRD patterns of AES biochar, Cu_2_O, and C-Cu. HR-TEM images of C-Cu with (**e**) Cu_2_O and (**f**) Cu^0^ planes. (**g**) SEM image of AES biochar. (**h**) N_2_ sorption isotherms of C-Cu and AES biochar. Inset: Pore size distribution.

The microstructure and chemical components of the *Artemia* egg shell biochar-hosted nano-Cu_2_O/Cu^0^ (C-Cu) is shown in Figure 1. 

The C-Cu sample possessed a rough surface (Figure 1a) and the length and width were around 2–3 μm (Figure 1b). Fine particles can be observed in the TEM images in Figure 1b,c. In the XRD pattern in Figure 1d, the AES biochar is mainly amorphous carbon, which is also shown in the C-Cu sample. The characteristic lines of Cu_2_O (JCPDS No. 65-3288) exposed well-crystalized Cu_2_O in the commercial cuprous oxide and C-Cu, while Cu^0^ (JCPDS No. 04-0836) was only detected in the C-Cu. The particle sizes of the Cu_2_O and Cu^0^ in the C-Cu calculated from Scherrer’s formula were 36 nm and 35 nm, respectively. The lattices in the HR-TEM images clearly present Cu_2_O (111) and Cu^0^ (111) planes in Figure 1e,f. As listed in Table 1, the Brunauer–Emmett–Teller (BET) specific surface area and pore volume of C-Cu decreased after the dispersion of Cu species. However, the micropore volume and the average Barrett–Joyner–Halenda (BJH) pore size were comparable to the AES biochar. The AES biochar showed a micro–meso–macro pore distribution (Figure 1h inset) with the most probable pore size of 4.8 nm, in accordance with the desorption hysteresis typical for mesoporous materials (Figure 1h). However, the mesopore and macropore volumes of the C-Cu were about 50% of those of the AES biochar, indicating that the dispersed nanoparticles of Cu_2_O and Cu^0^ blocked some of the mesopores and macropores of the C-Cu. Thus, the hierarchical porous structure of the AES (Figure 1g) remained in the C-Cu, with the pores confined to the nanoparticle dispersion. The characterization results demonstrated that nanoparticles of Cu_2_O and Cu^0^ were loaded successfully on the AES biochar using the abovementioned synthesis method. The content of Cu in the C-Cu was 117.8 mg/g.

### 3.2. Iodine Removal Efficiency by C-Cu

**Figure 2:**
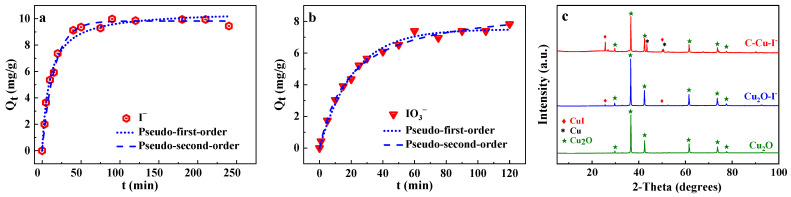
Data on the adsorption of (**a**) iodide ions and (**b**) iodate ions onto C-Cu fitted by kinetic models: C_0_ = 10 mg/L, adsorbents 1.0 g/L, 25 °C, pH = 7.0 ± 0.2. (**c**) XRD patterns of Cu_2_O, Cu_2_O-I^−^, and C-Cu-I^−^.

**Figure 3:**
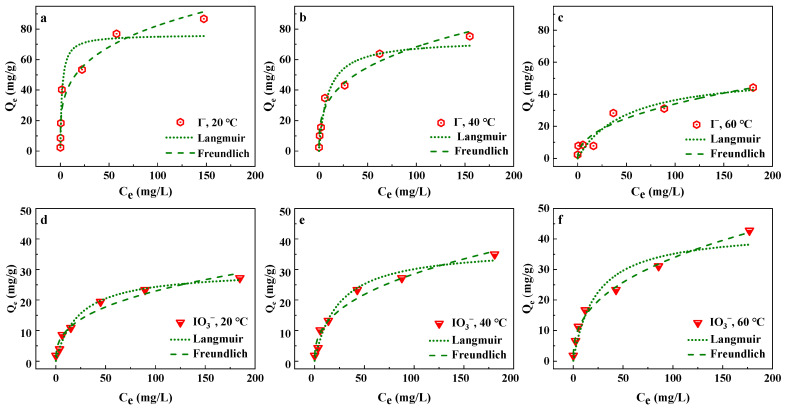
Isotherm adsorption of iodide ions onto C-Cu at (**a**) 20 °C, (**b**) 40 °C, and (**c**) 60 °C and of iodate ions at (**d**) 20 °C, (**e**) 40 °C, and (**f**) 60 °C. C_0_ = 10 mg/L, C-Cu dose 0.5 g/L, pH = 7.0 ± 0.2.

The iodine removal efficiency was evaluated using the removal kinetics and isotherm adsorption of the C-Cu, and the experimental data and fitting results are illustrated in Figure 2 and Figure 3, respectively. Pseudo-first order and pseudo-second order models are shown in Equations (1) and (2), respectively [28]. 

Pseudo-first order model:

(1) $$Q_{t}{=Q}_{e}{(1-e}^{-k_{1}t})$$

Pseudo-second order model:

(2) $$Q_{t}=\frac{Q_{e}^{2}k_{2}t}{1+Q_{e}k_{2}t}$$

Here, *Q_t_* (mg/g) is the adsorbed amount of iodide ions or iodate ions at a certain time *t* (min); *Q_e_* (mg/g) is the adsorption capacity of the iodide ions or iodate ions at equilibrium; *k*_1_ (1/min) and *k*_2_ (g⸱mg^−1^⸱min^−1^) are the kinetic rate constants of the pseudo-first order and pseudo-second order models, respectively.

It is clear from the kinetic data of the removal of iodide ions by C-Cu in Figure 2a that the rate of iodide ion adsorption was very rapid, reaching 50% of the maximum adsorption capacity at 10 min and the adsorption equilibrium at 40 min. In Figure 2b, the iodate ion adsorption capacity reached half of its maximal capacity at 20 min, and then the adsorption rate slowed and equilibrated at 60 min. The adsorption of iodide and iodate ions onto C-Cu was fitted using pseudo-first order and pseudo-second order models, and the results more consistent with the pseudo-second order kinetic model (Table 2). This kinetic fitting results explained the external and internal diffusion and adsorption of iodide and iodate anions onto the active sites of the porous C-Cu [28]. 

The Langmuir and Freundlich models shown in Equations (3) and (4) are used to fit the isotherm adsorption data, respectively [29].

Langmuir isotherm model:

(3) $$Q_{e}=\frac{Q_{m}K_{L}C_{e}}{1+K_{L}C_{e}}$$

Freundlich isotherm model:

(4) $$Q_{e}=K_{F}C_{e}^{\frac{1}{n}}$$

Here, *Q_e_* (mg/g) and *C_e_* (mg/L) are the adsorbed amount and concentration of iodide ions or iodate ions at equilibrium; *Q_m_* (mg/g) is the maximum adsorption capacity of the Langmuir model; *K_L_* (L/mg) is the ratio of the adsorption and desorption rates; *K_F_* (L^1/*n*^⸱mg^(1−1/*n*)^⸱g^−1^) and *n* are the Freundlich model constants.

In Figure 3, the maximum adsorption capacity values of the iodide ions decreased with the increase in temperature, indicating that low temperatures were more favorable for C-Cu to adsorb iodide ions. At 20 °C, the maximum adsorption capacity reached 86.8 mg/g. Regarding the adsorption of iodate ions onto the C-Cu, the temperature presented opposite effects on the capacity compared with the iodide ions; the maximum adsorption capacity values increased with the increases in temperature to 29.8 mg/g, 37.4 mg/g, and 43.1 mg/g at 20 °C, 40 ℃, and 60 °C, respectively. Table 3 shows the regression coefficients of the iodine adsorption data onto C-Cu fitted by the Langmuir and Freundlich isothermal models, where the data are more consistent with the Freundlich model.

The removal mechanism of iodine by C-Cu was extrapolated using XRD patterns of the C-Cu before and after iodine adsorption, with Cu_2_O used as for comparison. The Cu_2_O and Cu^0^ crystal diffraction peaks were present in the C-Cu. After the adsorption of iodide ions, the C-Cu showed new diffraction peaks at 2*θ* = 25.6°, 50.0°, and 67.5°, corresponding to the (111), (311), and (311) crystal planes of *γ*-CuI (JCPDS No. 06-0246), respectively, which proved the generation of CuI. The stronger intensity of the diffraction peak of *γ*-CuI in the XRD pattern of C-Cu-I compared to Cu_2_O after I^−^ absorption further indicated the stronger I^−^-binding ability of C-Cu.

Compared with the iodine removal materials listed in Table 4, the as-prepared *Artemia* egg shell biochar-hosted nano-Cu_2_O/Cu^0^ showed effective iodine removal performance in terms of the adsorption kinetics and maximum actual adsorption capacity (Q_a_) [30,31,32,33]. The hierarchical porous structure of the *Artemia* egg shell biochar facilitates the faster mass transfer of iodide and iodate anions onto the C-Cu surface, leading to equilibrium being achieved much quicker at 40 min. Moreover, the hierarchical porous structure promotes nano-Cu_2_O and Cu^0^ dispersion on the AES biochar surface, which provides abundant active sites for iodide or iodate ion sorption. Thus, high maximal adsorption capacities are achieved for iodide and iodate ions.

### 3.3. Effect of Solution pH on Iodine Removal

**Figure 4:**
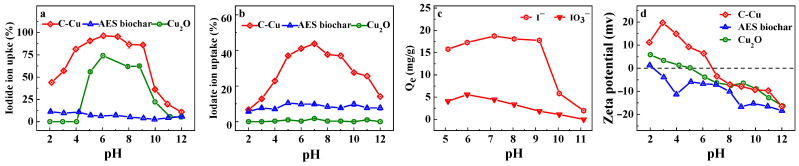
Effect of the solution pH on (**a**) iodide anion and (**b**) iodate anion removal rates by Cu_2_O, AES biochar, and C-Cu. (**c**) Effect of the solution pH on iodide ion and iodate ion adsorption onto C-Cu. C_0_ = 10 mg/L, adsorbent dosage 0.5 g/L, 25 °C. (**d**) Zeta potentials of Cu_2_O, AES biochar, and C-Cu.

The iodine species vary at different solution pH levels, mainly occurring as iodide anions and iodate anions [13]. The effects of the solution pH on iodide and iodate anions are illustrated in Figure 4. In the experiment, the AES biochar removed 2.6% to 10.5% of the iodine as both iodide and iodate anions in the pH range of 2.1 to 12.0, while the C-Cu achieved good iodine removal at neutral pH. Over a wide pH range of 4.0 to 9.1, C-Cu maintained iodide ion removal rates higher than 81.5%, with maximum removal rates of around 96.4% at pH = 6.1 and 7.2. With increasing pH levels of 10.0, 10.9, and 12.0, the rate of iodide ion removal by C-Cu rapidly declined, reaching only 10.9% at 12.0. This pattern was consistent with the iodide ion removal by Cu_2_O, but due to the wider pH range and higher iodine uptake rates, the C-Cu was more efficient for iodine removal. The removal rate was related to the nanoparticle stability at different solution pH levels, as shown in Table 5. In acid solutions, the nanoparticles on the C-Cu leached by up to 26.8% at pH 3.1, which decreased the iodide and iodate ion adsorption and removal rates at acidic pH values. On the other hand, the nanoparticles remained stable in near-neutral to alkaline solutions, with a leaching rate of 2.0% at pH 7.2. Moreover, the zeta potentials of the AES biochar, Cu_2_O, and C-Cu, as illustrated in Figure 4c, can be used to explain the iodide ion removal efficiency. All sorbents showed decreased seta potentials with increasing pH values, but the AES biochar showed negative zeta potentials across the majority of the experimental pH range, which increased the repulsion towards the iodide anions on the biochar surface. In contrast, both Cu_2_O and C-Cu showed positive zeta potentials in the acidic to near-neutral pH range, with zero charge potentials of 5.10 and 6.75 for Cu_2_O and C-Cu, respectively. Positive potentials promoted high iodide ion removal efficiency by C-Cu by enhancing the electrostatic attraction to the iodide anions. The lower removal rates of iodide ions by Cu_2_O at pH values of 2.0 to 4.0 in Figure 4a might be attributed to the instability of Cu_2_O in acid solutions, which was mitigated by the pore confinement of Cu_2_O on C-Cu and led to an iodide ion removal rate of around 44.1% at pH = 2.0. Although the zeta potential partly indicated how the pH of the solution affected the removal of iodide ions, the electrostatic effect was not the only reason for the iodide ion removal. Even at lower pH values, Cu_2_O showed low iodate anion removal efficiency, despite showing positive zeta potentials. In comparison, C-Cu demonstrated removal rates of 43.7% for iodate ions in an iodate solution (Figure 4b), and 22.3% for iodate ions and 93.7% for iodide ions in an iodide and iodate solution, both at pH = 7.0 ~ 7.1. In addition to the electrostatic effect, a more complicated mechanism of iodine removal by C-Cu was indicated by the different affinities of the iodide and iodate ions on Cu_2_O and C-Cu.

### 3.4. High Selectivity of Iodide and Iodate Ions against Competitive Anions

**Figure 5:**
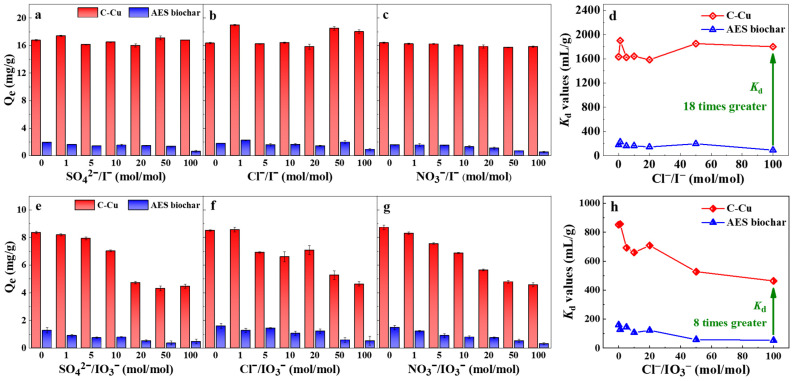
Effects of competitive ions on the removal of iodide and iodate anions: (**a**–**c**) I^−^ adsorption against SO_4_^2−^ Cl^−^, and NO_3_^−^; (**d**) *K*_d_ value of I^−^ against Cl^−^; (**e**–**g**) IO_3_^−^ adsorption against SO_4_^2−^, Cl^−^, and NO_3_^−^; (**h**) *K*_d_ value of IO_3_^−^ against Cl^−^; adsorbents 0.5 g/L, 25 °C, pH = 7 ± 0.2.

As shown in Figure 5, C-Cu demonstrated remarkable selectivity towards both iodide and iodate ions against competing anions (Cl^−^/NO_3_^−^/SO_4_^2−^) at high concentrations. Neither the competing anion species nor the concentration significantly weakened the adsorption of iodide ions onto C-Cu in the experimental. The distribution coefficient *K*_d_ increased by 18-fold when compared to the AES biochar, indicating that C-Cu has an extraordinarily high affinity for iodide ions that is not only brought about by electrostatic effects. The adsorption selectivity of C-Cu for iodide ions increased by around 20% over that of Cu_2_O, which should also be mentioned because it suggested that the affinity sites were used to their greater potential. The hierarchical porous structure of the AES biochar promoted the in situ generation of nano-Cu_2_O and Cu^0^ and uniform nanoparticle dispersion on the porous surface, which was attributed to the presence of high-affinity sites for iodide ions. The high selectivity of C-Cu towards iodine was also exhibited by the iodate ion adsorption. Even while the adsorption of C-Cu towards the iodate ions was somewhat reduced by the three competing anions with high concentrations, the *K*_d_ value of the iodate ions against 100-fold Cl^−^ (mol/mol) for the C-Cu was still 8 times higher than that of the AES biochar. Therefore, C-Cu offered superior iodine removal performance for natural water sources where large amounts of anions were present.

### 3.5. Continuous Adsorption Performance

**Figure 6:**
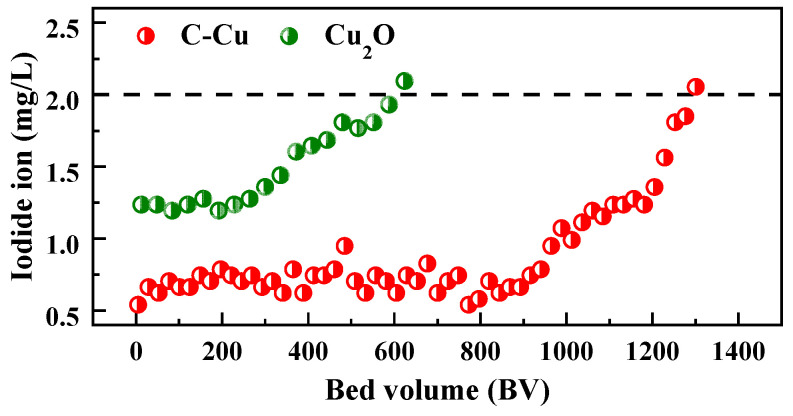
Column adsorption of iodide ions onto Cu_2_O and C-Cu. C_0_ = 2.0 mg/L, adsorbents 1.5 g, 25 °C, pH = 7.0 ± 0.2, Cl^−^ = 150 mg/L, SO_4_^2−^ = 50 mg/L, NO_3_^−^ = 50 mg/L, F^−^ = 50 mg/L, ReO_4_^−^ = 10 mg/L, HCO_3_^−^ = 50 mg/L, 20 BV/h.

To further investigate the applicability of C-Cu in an engineering continuous flow treatment system, a fixed-bed column adsorption experiment was conducted, with commercial Cu_2_O powder used as a comparison, and the experimental data are shown in Figure 6. The concentration of iodide ions in the influent was 2.0 mg/L, and the concentration in the effluent of 941 BV remained stable at about 0.78 mg/L, which provided an iodide ion removal rate of about 61%. After this, the I^−^ concentration in the effluent started to rise and a breakthrough occurred when the effluent reached 1279 BV. The fixed-bed column packed with C-Cu processed more than four times as much water as the Cu_2_O column did, while also having a lower iodide ion concentration in the effluent, which was ascribed to the hierarchical porous structure of the C-Cu and pore-confined nanoparticles. The large water treatment volumes and high iodine removal performance rates under engineering conditions demonstrated the application potential of C-Cu for the deep treatment of iodine-containing wastewater. 

## 4. Conclusions

A novel adsorbent was synthesized via the in situ precipitation and reduction of Cu_2_O and Cu^0^ on a porous biomatrix of *Artemia* egg shell biochar. SEM, TEM, XRD, and BET analyses confirmed that approximately 35 nm of nano-Cu_2_O/Cu^0^ dispersed in the pores of the AES biochar. The C-Cu induced a rapid adsorption equilibrium for iodide and iodate ions. The adsorption isotherm experiments showed that the maximum actual adsorption capacities of the iodide and iodate ions by C-Cu reached 86.8 and 43.1 mg/g at pH = 7.0 ± 0.2, respectively. Moreover, for the iodide and iodate ions, the C-Cu achieved *K*_d_ values about 18 and 8 times greater than for the AES biochar against Cl^−^ at a 100-fold molar ratio. These excellent adsorption performance results were attributed to mechanisms including electrostatic interactions and precipitation. The continuous adsorption experiments demonstrated the potential of C-Cu for engineering applications. Overall, the rapid and selective adsorption of iodide and iodate ions onto C-Cu makes this nanocomposite attractive for efficiently removing radioactive iodine from water.

## Figures and Tables

**Table 1:** Specific surface area, pore volume, and pore size values of AES biochar and C-Cu.

Adsorbents	Specific Surface Area (m^2^/g)	Pore Volume (cm^3^/g)	Micropore Volume (cm^3^/g)	BJH Average Pore Size (nm)
**AES biochar**	13	0.045	0.004	7.8
**C-Cu**	8	0.023	0.003	7.3

**Table 2:** Adsorption kinetic parameters of iodide and iodate ions on C-Cu.

	Pseudo-First Order			Pseudo-Second Order		
	*Q_e_* (mg/g)	*k*_1_ (1/min)	*R* ^2^	*Q_e_* (mg/g)	*k*_2_ (g⸱mg^−1^⸱min^−1^)	*R* ^2^
**I^−^**	9.8	0.0630	0.954	10.6	0.0097	0.987
**IO_3_^−^**	7.5	0.0467	0.993	9.1	0.0057	0.994

**Table 3:** Adsorption isotherm parameters of iodide and iodate ions on C-Cu.

Iodine	*T* (K)	Langmuir Model			Freundlich Model		
		*Q_m_* (mg/g)	*K_L_* (L/mg)	*R* ^2^	*K_F_* (L^1/*n*^⸱mg^(1−1/*n*)^⸱g^−1^)	*n*	*R* ^2^
**I** **^−^**	293	76.3	0.550	0.932	23.81	−0.269	0.944
	313	73.1	0.111	0.949	15.96	−0.316	0.974
	333	54.5	0.020	0.907	4.38	−0.445	0.921
**IO_3_** **^−^**	293	29.8	0.044	0.985	4.14	−0.372	0.968
	313	37.4	0.042	0.977	4.75	−0.390	0.972
	333	43.1	0.044	0.938	5.64	−0.389	0.994

**Table 4:** Iodide ion removal performance parameters of different adsorbents.

Adsorbents	Adsorption Conditions	Equilibrium Time (Min)	Q_a_ (mg/g)		Reference
			I^−^	IO_3_^−^	
**Ag@MIL-101**	C_0_ = 12.7–508 mg/L, pH = 7.0, dose 1.0 g/L	180	2.1	—	[30]
**Ag@Cu_2_O**	C_0_ = 2.5~38 mg/L, pH = 3~10, dose 1.0 g/L	180	25.4	—	[31]
**Cu_2_O-C**	C_0_ = 1~20 mg/L, pH = 7, dose 1.0 g/L	120	41.2	—	[32]
**Cu/Cu_2_O-LDH**	C_0_ = 0~240 mg/L, pH = 6.5, dose 1.0 g/L	265	137.8	—	[33]
**C-Cu**	C_0_ = 1~200 mg/L, pH = 4~10, dose 0.5 g/L	40 (I^−^), 60 (IO_3_^−^)	86.8	43.1	This work

**Table 5:** Cu leaching rates (%) in solutions with different pH values.

Solution pH	Cu leaching Rate (%)	Solution pH	Cu leaching Rate (%)
3.0	26.8	8.0	1.1
4.0	11.9	9.0	1.0
5.0	7.2	10.0	0.8
6.0	4.6	11.0	0.1
7.0	2.0	12.0	0.1

## Data Availability

The data presented in this study are available on request from the corresponding author.
